# Autoimmune brainstem encephalitis: Clinical associations, outcomes, and proposed diagnostic criteria

**DOI:** 10.1002/acn3.52273

**Published:** 2024-12-21

**Authors:** Michael Gilligan, Smathorn Thakolwiboon, Emma Orozco, Samantha Banks, Eoin P. Flanagan, Sebastian Lopez‐Chiriboga, Jan‐Mendelt Tillema, John R. Mills, Sean J. Pittock, Cristina Valencia Sanchez, Anastasia Zekeridou, Divyanshu Dubey, Andrew McKeon

**Affiliations:** ^1^ Department of Laboratory Medicine and Pathology Mayo Clinic Rochester Minnesota USA; ^2^ St Vincent's Hospital, University College Dublin Elm Park Dublin Ireland; ^3^ Department of Neurology Mayo Clinic Rochester Minnesota USA; ^4^ Department of Neurology Mayo Clinic Jacksonville Florida USA; ^5^ Department of Neurology Mayo Clinic Phoenix Arizona USA

## Abstract

**Objective:**

We describe neurologic phenotype, clinical associations, and outcomes in autoimmune brainstem encephalitis.

**Methods:**

Medical records of neural‐IgG positive autoimmune brainstem encephalitis patients diagnosed at Mayo Clinic (January 1, 2006–December 31, 2022) were reviewed.

**Results:**

Ninety‐eight patients (57 male) were included. Median age of symptom onset was 51 years (range, 8 months‐85 years). Frequent presenting features were ≥1: diplopia (80%), ataxia (78%), dysarthria (68%), vestibulocochlear symptoms (67%), dysphagia (61%), nausea/vomiting (42%), and facial weakness (32%). Altered mental status (11%) was uncommon. Neural antibodies detected were as follows: KLHL‐11 (26 patients), GAD65 (high titer, 12), ANNA‐1 (anti‐Hu, 8), ANNA‐2 (anti‐Ri, 8), Ma2 (7), IgLON‐5 (6), AQP4 (6), MOG (4), glycine receptor (4), GQ1B (4), PCA‐1 (anti‐Yo, 4), DPPX (2), neurochondrin (2), neurofilament (2), NMDA‐R (2), AGNA‐1 (SOX‐1, 1), ANNA‐3 (DACH1, 1), amphiphysin (1), CRMP‐5 (1), ITPR‐1 (1), PCA‐Tr (DNER, 1), and PDE10A (1). Cancer was identified in 55 patients: germ cell (23 patients; 3 extra‐testicular), ductal breast adenocarcinoma (8), small cell carcinoma (6, lung 4), adenocarcinomas (6), neuroendocrine carcinoma (3), hematologic (2), squamous cell (2), and other (7). Median modified Ranking score (mRS) at last follow‐up was 3 (range, 0–6). Factors associated with poor outcome included abnormal brain MRI, bulbar symptoms, and elevated CSF IgG index. Kaplan–Meier analysis revealed faster progression to wheelchair in patients who were immunotherapy refractory and with elevated CSF IgG index. Diagnostic criteria for autoimmune brainstem encephalitis (definite and probable) are proposed.

**Interpretation:**

Autoimmune brainstem encephalitis is a distinct clinical subphenotype of autoimmune encephalitis. Abnormal brain MRI, bulbar symptoms, and elevated CSF‐IgG index associate with poor outcome.

## Introduction

Autoimmune brainstem encephalitis (rhombencephalitis) is typically characterized by subacute onset gait difficulties, oculomotor abnormalities, vestibulocochlear dysfunction, facial weakness, and bulbar symptoms. This disorder may be considered isolated (when only brainstem and cerebellum are affected) or multifocal when additional symptoms referrable to other anatomic sites in the central or peripheral nervous systems are also present. This phenotype accounts for 12% of all autoimmune encephalopathies encountered in our clinical practice.^1^ Altered mental status, the cardinal feature of autoimmune encephalitis, is usually absent. Thus, patients with brainstem presentations are not sensitively captured by existing autoimmune encephalitis diagnostic criteria, which require altered mental status, working memory deficits, or psychiatric symptoms.^2^ While literature exists on individual autoimmune brainstem disorders defined by disease‐specific antibodies (e.g., brainstem encephalitis associated with antineuronal nuclear antibody [ANNA]‐1, also known as anti‐Hu, Ma2 or Kelch‐like protein‐11 [KLHL‐11]‐IgGs), there are limited data describing the neurologic, serologic, and oncologic profiles of patients who present with this clinical phenotype.^3^, ^4^, ^5^ Herein, we define these parameters, determine factors associated with poor outcome, and propose diagnostic criteria for patients with autoimmune brainstem encephalitis using a large cohort from a tertiary referral center.

## Methods

### Standard protocol approval and patient consent

The retrospective cohort study was approved by the Mayo Clinic Institutional Review Board (IRB #21‐001297). All patients included consented to use of their medical record for research purposes.

### Patient inclusion and data collection

Patients were identified by interrogating the electronic medical record system (Mayo Clinic Rochester, MN; Scottsdale, AZ; Jacksonville, FL) between January 1, 2005, to December 31, 2022, for patients diagnosed with “brainstem encephalitis” or “rhombencephalitis.” The initial search returned 714 patients with research authorization documented. Of 714 patients, 633 patients were excluded from the study due to one of the following reasons: nonautoimmune final diagnosis, unclear final diagnosis, autoimmune CNS disorder without prominent brainstem involvement, likely autoimmune brainstem encephalitis without neural antibody biomarker, or patient assessment outside study timeframe. Seventeen additional patients were identified by interrogating pre‐existing Mayo Clinic databases of autoimmune neurologic disorders (MOG and neurochondrin autoimmunity).^1^, ^6^, ^7^ Patients were included if autoimmune brainstem encephalitis/rhombencephalitis was diagnosed by a neurologist at our institution, in addition to the presence of a pertinent neural antibody biomarker (Fig. S1).

Patients were defined as having isolated brainstem encephalitis if symptoms and signs referred only to brainstem and cerebellum; cases with prominent brainstem symptoms in addition to clinical symptoms referrable to cerebral cortex, spinal cord, or peripheral nervous system were defined as brainstem encephalitis accompanying a multifocal disorder. Patients whose first presentation did not include a brainstem syndrome were not included (e.g., MOGAD presenting initially with optic neuritis and developing a brainstem syndrome subsequently). Patients were either referred for evaluation at the Mayo Clinic as an outpatient or assessed initially as an inpatient at a Mayo Clinic hospital.

Neural antibodies considered pertinent were as follows: amphiphysin; anti‐glial nuclear antibody (AGNA)‐1 (SOX‐1); ANNA‐1, −2 (anti‐Ri) and‐3 (DACH1); aquaporin‐4 (AQP4), collapsin‐response mediator protein (CRMP)‐5; contactin‐associated protein 2 (CASPR2); dipeptidyl‐peptidase‐like protein (DPPX); glutamic acid decarboxylase 65 kDa isoform (GAD65) antibody (high titer in the serum [>20 nmol/L] or any titer detected in the CSF); glial fibrillary acidic protein (GFAP); glycine receptor; GQ1B; GTPase regulator associated with focal adhesion kinase (GRAF)‐1; IgLON‐5; inositol 1,4,5‐triphosphate receptor (ITPR)‐1; KLHL‐11; leucine‐rich glioma‐inactivated [LGI1] antibody; neurochondrin; neuronal intermediate filament (NIF; alpha internexin or neurofilament light or heavy chain), only in CSF; N‐methyl‐D‐aspartate receptor (NMDA‐R), only in CSF; Ma2; myelin oligodendrocyte glycoprotein (MOG); Purkinje cell cytoplasmic antibody (PCA)‐1 (anti‐Yo); PCA‐Tr; phosphodiesterase 10A (PDE10A). Positive neural antibodies were defined as “intracellular” when the antigen target was subcellular, or “extracellular” when the target antigen consisted of a cell‐surface or transmembrane protein. We excluded antibodies with limited specificity for neurologic disease. These were as follows: calcium channel antibodies, low‐positive glutamic acid decarboxylase [GAD] 65 antibody in serum (<20 nmol/L), NMDA‐R, neuronal intermediate filament [NIF], or glial fibrillary acidic protein [GFAP]‐IgGs detected in serum only. Variables extracted from the electronic medical record included: age, sex, clinical features, comorbidities and medical history, cancer history, MRI and CSF results, immunotherapies utilized, modified Rankin score (mRS) at last follow‐up. Patient data were stored in a password‐protected database accessible by two investigators (MG and AM).

Bulbar symptoms were defined as presence of clinical features localizing to the medulla (e.g., dysphagia). Abnormal brain MRI was defined as ≥1 abnormalities of the following regions of cerebellum, brainstem, or cerebrum: signal change reported as abnormal (excluding changes consistent with small vessel disease), enhancing MRI lesions, or atrophy out of proportion for age. Inflammatory CSF was defined as the presence of ≥1: pleocytosis, elevated IgG index, elevated kappa free light chains, or CSF‐exclusive oligoclonal bands (elevated CSF protein was considered an insufficiently specific marker of CSF inflammation and not included). Poor outcome was defined by mRS ≥4 at last follow‐up. mRS was used as the outcome measure given ease of applicability in a retrospective cohort, and widespread use and understanding of mRS scores in the neurologic community; mRS ≥4 was considered poor outcome in this cohort because of the high frequency of gait disorder with severe cases defined by residual gait dependence. Subjective treatment response was defined as patient‐ or physician‐reported improvement without improvement in mRS score. Objective treatment response was defined as improvement in mRS score following treatment initiation. Refractory response to immunotherapy refers to patients who had neither an objective or subjective response to a trial of immunotherapy.

### Statistical analysis

All statistical calculations were performed using IBM SPSS Statistics version 28 (IBM Corp, Armonk, NY). Categorical variables were analyzed using frequency and percentage, while continuous variables were described using median and range. Chi‐square tables were used to assess univariate factors associated with poor outcome. Variables found to potentially affect outcomes (*p* < 0.1) from univariate analysis were included as candidate variables in multivariate analysis to determine factors associated with poor outcome. For overlapping variables (e.g., gaze palsy and diplopia), the more significant variable was chosen in order to avoid multicollinearity. Intergroup categorical data were compared using the Fisher exact test or chi square, as appropriate (*p* < 0.05 was considered significant). Time to wheelchair dependence was analyzed using a Kaplan–Meier survival analysis. Multivariate Cox regression analysis was performed to determine factors independently associated with progression to wheelchair dependence (*p* < 0.05 was considered significant).

## Results

Ninety‐eight patients with a diagnosis of autoimmune brainstem encephalitis and pertinent neural antibody positivity were included.

### Demographic data

Of 98 patients, 57 (58%) were male. The median age of symptom onset was 51 years (range 8 months – 85 years). Five patients (5%) were pediatric (<18 years) at symptom onset. Symptom onset was subacute (>1 week and <3 months) in 80 patients (82%). An infectious prodrome (defined as low‐grade fever, or flu‐like illness preceding neurologic symptoms) was present in 21 patients (21%). A personal history of autoimmunity was reported in 24 patients (25% [non‐neurologic autoimmunity, 22 patients; neurologic autoimmunity, 2 patients]). A list of autoimmune conditions is provided in Table S6. Among these patients, the most frequent neural antibodies detected were as follows: GAD65 (7), AQP4 (2), ANNA‐1 (2), ANNA‐2 (2), glycine receptor (2), KLHL‐11 (2), and GQ1B (2). No patient had a medication history of immune checkpoint inhibitor use.

### Main neurologic features

Isolated brainstem encephalitis occurred in 52 patients (53%); 46 had a multifocal neurologic disorder. Overall, the most frequent clinical features were diplopia (78 patients, 80%), coexisting ataxia (76, 78%), vestibulocochlear dysfunction (66, 67%), dysarthria (67, 68%), and dysphagia (60, 61%). Higher cortical features were infrequent with only 11 patients (11%) experiencing alteration in mental status, 13 (13%) with short‐term memory loss and 13 had seizures. Six patients (6%) had reduced level of consciousness; 5 (5%) had psychiatric symptoms. An expanded breakdown of clinical features is included in Table 1 and Table S1.

**Table 1:** Demographic and clinical characteristics.

Demographic details	No. (%)
Total patients	98
Male sex	57 (58)
White, non‐Hispanic	81 (of 92 with data available [88%])
African American	9 (of 92 with data available [10%])
Hispanic	1 (of 92 with data available [1%])
Asian	1 (of 92 with data available [1%])
Age of symptom onset	51 (range, 8 months‐85 years)
Subacute onset	80 (82)
Infectious prodrome	21 (21)
Personal history autoimmunity	24 (25)
Immune checkpoint inhibitor exposure	0 (0)

Supratentorial symptoms	No. (%)
Short‐term memory loss	13 (13)
Altered mental status	11 (11)
Decreased consciousness	6 (6)
Psychiatric symptoms	5 (5)
Seizures	13 (13)

Brainstem signs and symptoms	No. (%)
Diplopia	78 (80)
Nystagmus	68 (69)
Dysarthria (cerebellar or bulbar)	67 (68)
Vestibulocochlear	66 (67)
Bulbar	64 (65)
Dysphagia	60 (61)
Ophthalmoplegia	42 (43)
Internuclear ophthalmoplegia	6 (6)
Nausea/vomiting	41 (42)
Facial weakness	31 (32)
Ptosis	26 (27)
Hearing loss	23 (24)
Myelopathy	20 (20)
Tongue weakness	11 (11)
Altered facial sensation	11 (11)
Palatal weakness	7 (7)
Respiratory failure	5 (5)
Laryngospasm	4 (4)
Cardiac arrest	3 (3)
Hiccups	3 (3)
SCM/Trapezius weakness	2 (2)

SCM, sternocleidomastoid.

### Movement disorders

Eighty‐six patients (88%) had a movement disorder, with ataxia being the most frequent (76, 78%). Other movement disorders included dystonia (17 patients, 17% [of whom 9 patients had jaw dystonia]), postural instability (16, 16%) and parkinsonism (8, 8%), opsoclonus‐myoclonus syndrome (6, 6%); stiff person syndrome (5, 5%), laryngospasm (4, 4%), palatal myoclonus (4, 4%), and chorea (3, 3%) (Table S1).

### Sleep disorders

Sleep disorders were prevalent among the cohort (28 patients, 29%) and included central sleep apnea (12, 12%), insomnia (10, 10%), REM sleep disorder (10, 5 of whom were IgLON5‐IgG positive), hypersomnia (6, 6%), and narcolepsy/cataplexy (3, 2 of whom had Ma2‐IgG).

### Neural antibodies

**Figure 1:**
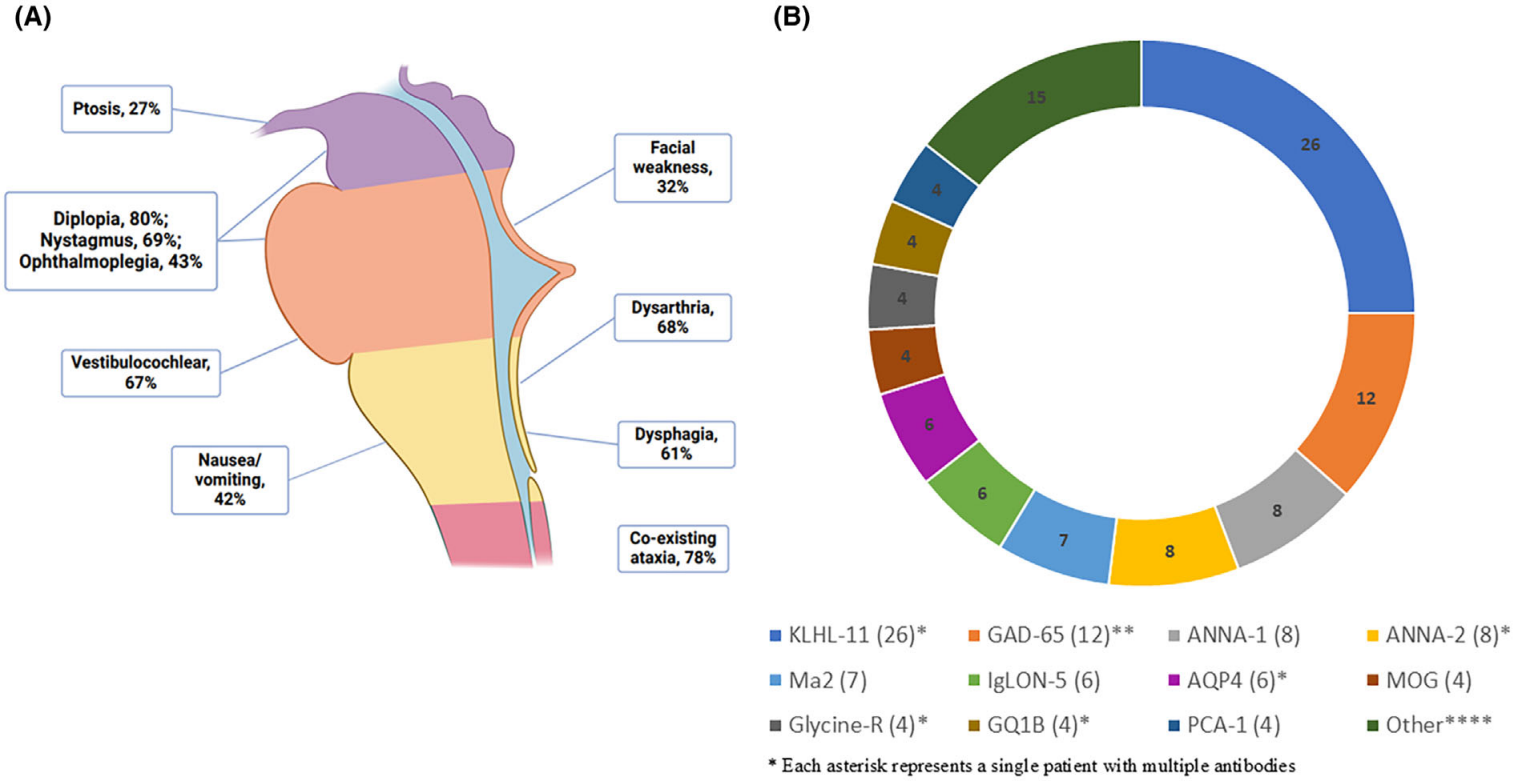
(A) Frequent clinical features associated with autoimmune brainstem encephalitis (B) Pie chart demonstrating neural antibody frequency among patients with autoimmune brainstem encephalitis. AGNA, anti‐glial neuronal nuclear antibody; ANNA, antineuronal nuclear antibody; AQP4, aquaporin‐4; CRMP, collapsin‐response mediator protein; DPPX, dipeptidyl aminopeptidase‐like protein; GAD, glutamic acid decarboxylase; ITPR, inositol 1,4,5‐triphosphate receptor; KLHL‐11, Kelch‐like protein 11; MOG, myelin oligodendrocyte glycoprotein; NIF, neuronal intermediate filament; NMDA‐R, N methyl D aspartate receptor; PCA, Purkinje cell cytoplasmic antibody; PDE10A, phosphodiesterase 10A.

By definition, all patients were positive for an antibody with high specificity for autoimmune neurologic disease in either serum (83 patients, [of 95 with data available, 85%]), CSF (56, [of 95 with data available, 59%]), or both (47, [of 95 with data available, 49%]). The most frequently identified neural antibody was KLHL‐11‐IgG (*n* = 26 [1 was positive for both KLHL‐11 and ANNA‐2 IgGs]), followed by GAD‐65‐IgG (*n* = 12 [1 had GAD‐65 coexisting with glycine receptor‐IgG; 1 had GAD‐65 coexisting with NMDA‐R and GQ1B‐IgGs]), ANNA‐1 (*n* = 8), and ANNA‐2 (*n* = 8 [1 had ANNA‐2 coexisting with KLHL‐11‐IgGs]). The relative frequency of neural antibodies is shown in Figure 1 and a complete list in Table S2. Almost all patients (93, 95%) had a single neural antibody detected in serum or CSF; multiple neural antibodies were present in 5 patients (5%). Seventy‐three patients (74%) were positive for an antibody directed against an intracellular antigen.

Eighteen patients with both specimen types tested were positive in serum only (KLHL‐11, 5; ANNA‐1, 2; Ma2, 2; IgLON5, 2; AQP4, 2; MOG, 2; AGNA‐1, 1; GAD65, 1; glycine‐R, 1), and 5 were positive in CSF only (KLHL‐11, 2; glycine receptor, 1; IgLON‐5, 1; Ma2, 1). A complete breakdown of clinical details for patients with multiple neural antibodies are provided in Table S3.

### Imaging

**Figure 2:**
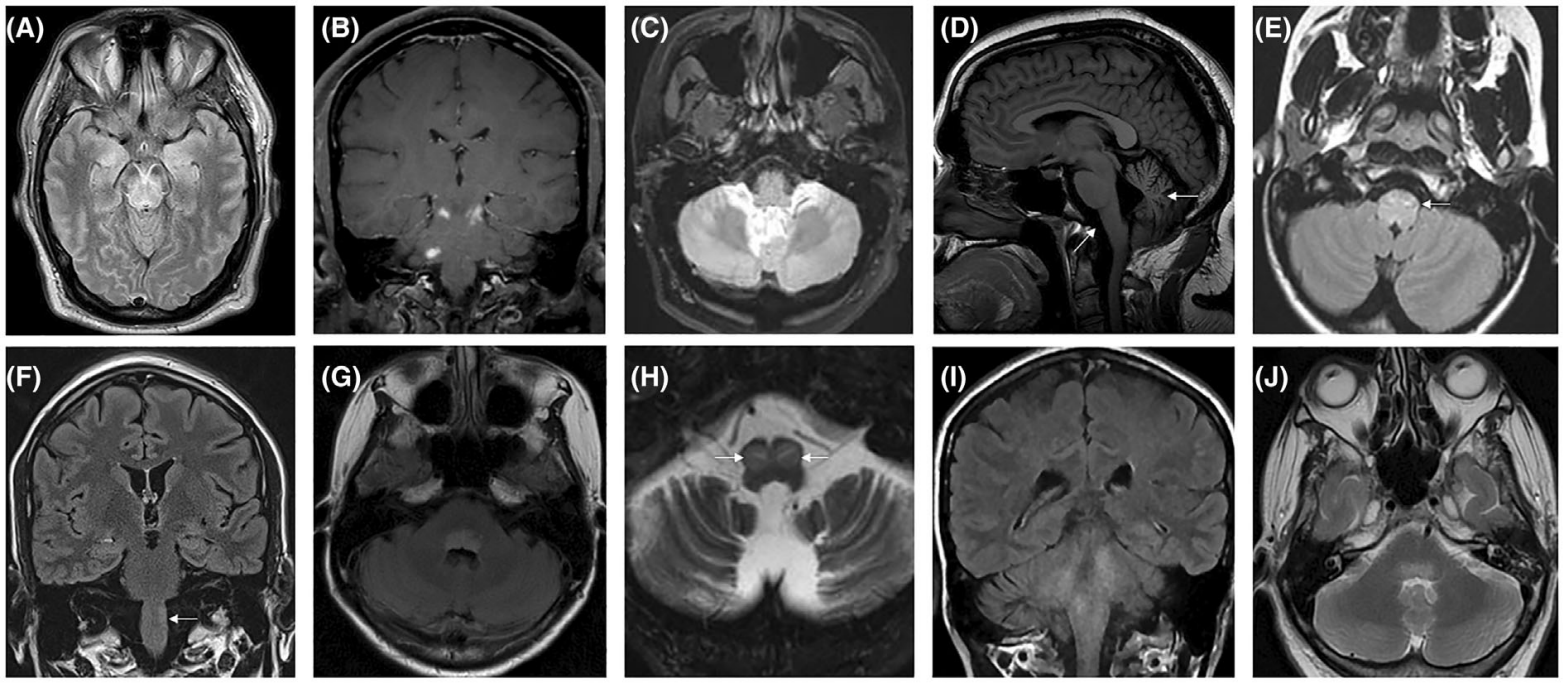
Selection of MRI abnormalities from patients with autoimmune brainstem encephalitis. (A) Axial T2 FLAIR image demonstrating midbrain signal change and bilateral mesial temporal lobe signal changes in a 46‐year‐old male with subacute onset ptosis, ophthalmoplegia and ataxia, positive for Ma2‐IgG. (B) Coronal gadolinium‐enhancing lesions affecting brainstem and cerebellum in 27‐year‐old female with MOGAD. (C) Axial T2 FLAIR signal change of cerebellum in a 50‐year‐old male with sensorineural hearing loss, diplopia, vertigo, and cerebellar ataxia and positive for KLHL11‐IgG. (D) Sagittal T2 image demonstrating severe brainstem and cerebellar atrophy (arrows) in a 20‐year‐old male with diplopia and ataxia, positive for ITPR‐1‐IgG. (E) Axial T2 FLAIR signal change involving the medulla in 10‐year‐old boy with GAD65‐IgG‐associated brainstem encephalitis. (F) Coronal T2image demonstrating signal change in left medulla (arrow) of 28‐year‐old male with autoimmune brainstem encephalitis associated with Ma2‐IgG and embryonal cell carcinoma of testis. (G) Axial T2 FLAIR image demonstrating signal change in dorsal pons in a 53‐year‐old female with AQP4 + NMOSD. (H) Axial T2 signal change in the medullary olives consistent with bilateral hypertrophic olivary degeneration in a 44‐year‐old male with palatal myoclonus, positive for KLHL‐11 IgG. (I) Coronal T2 FLAIR signal change affecting medulla, pons and cerebellum in 48‐year‐old woman with autoimmune brainstem encephalitis associated with NMDA‐R, GAD65 and GQ1B‐IgGs. (J) Axial T2 signal change in the dorsal pons in 45‐year‐old male with KLHL‐11‐IgG‐associated rhombencephalitis and germ cell tumor of testis.

Representative MRI images are shown in Figure 2. Sixty‐two patients (63%) had an abnormal brain MRI (defined as signal change reported as abnormal, enhancing lesions, or atrophy out of proportion for age). Of these 62 patients, the most common finding was brainstem T2 hyperintensity (*n* = 30, 31%); followed by cerebellar atrophy (*n* = 27, 28%), cerebellar T2 hyperintensity (*n* = 9, 9%), brainstem atrophy (*n* = 6, 6%), and hypertrophic olivary degeneration (*n* = 3, 3%; KLHL‐11‐IgG [2], GAD65‐IgG [1]). Further details on MRI imaging abnormalities are presented in Table 2.

**Table 2:** MRI, CSF, and oncological features.

MRI	No. (%)
Abnormal MRI Brain	62 (63)
Brainstem signal change	30 (31)
Gadolinium‐enhancing brain or spinal cord lesion	24 (of 96 with data available [25%])
Cerebellar atrophy	27 (28)
Cerebellar signal change	9 (9)
Spinal cord lesion	7 (of 70 tested [10%])
Brainstem atrophy	6 (6)
Hypertrophic Olivary Degeneration	3 (3)
Brainstem lesion extending into spinal cord	4 (of 30 with brainstem lesion [13%])

CSF	No. (% or range)
Inflammatory CSF	67 (of 88 tested [76%])
CSF‐exclusive oligoclonal bands (>2)	51 (of 80 tested [64%])
CSF pleocytosis (>5 cells/μl)	38 (of 88 tested [43%])
Median cell count	13 (6–392)
Lymphocytic pleocytosis	26 (68% [of patients with pleocytosis])
Elevated IgG index (>0.85)	28 (of 74 tested [38%])
CSF Kappa Free Light Chain (>0.1 mg/dL)	6 (of 8 tested [75%])
Elevated CSF protein (≥35 mg/dL)	51 (of 87 tested [59%])

Oncological associations	No. (%); neural antibody association (*n*)
Cancer present	55 (56)
>1 cancer detected	2 (2)
Neurologic disorder prior to cancer detection	38 (69)
Testicular germ cell	20 (20); KLHL‐11 (14); KLHL11 and ANNA‐2 (1); Ma2 (4); glycine receptor (1)
Ductal carcinoma in situ (breast)	8 (8); ANNA‐2 (5); amphiphysin (1); AQP4 (1); GAD‐65 (1)
Small cell (pulmonary, 4; and extrapulmonary, 2)	6 (6); ANNA‐1 (4); ANNA‐3 and NIF (1); CRMP‐5 (1)
Adenocarcinoma (lung, 3; breast,1; ovary, 1; prostate, 1)	6 (6); KLHL‐11 (2); AGNA‐1 (1); ANNA‐2 (1); GAD‐65 (1); PCA‐1 (1)
Seminoma (extra‐testicular)	3 (3.1); KLHL‐11 (3)
Neuroendocrine	3 (3.1); GAD‐65 (1); Ma2 (1); NIF (1)
Hematologic	2 (2); PCA‐1 (1); glycine receptor (1)
Squamous cell (tonsil 1, tongue 1)	2; Ma2 (2)
Leiomyosarcoma	1; ANNA‐1 (1)
Neuroblastoma	1; ANNA‐1 (1)
Cervical (histology unknown)	1; ANNA‐2 (1)
Thymoma	1; ANNA‐1 (1)
Serous, fallopian tube	1; PCA‐1 (1)
Ovarian Brenner tumor	1; PCA‐1 (1)
Unknown histology	1; IgLON‐5 (1)

AGNA, anti‐glial nuclear antibody; ANNA, antineuronal nuclear antibody; AQP4, aquaporin 4; CRMP5, collapsin‐response mediator protein‐5; CSF, cerebrospinal fluid; GAD, glutamic acid decarboxylase; KLHL‐11, kelch‐like protein 11; NIF, neuronal intermediate filament; PCA, Purkinje cell cytoplasmic antibody.

### Cerebrospinal fluid

Sixty‐seven patients (of 88 tested [76]%) had an inflammatory CSF (defined as the presence of ≥1: pleocytosis, elevated IgG index, elevated kappa free light chains, and CSF‐exclusive oligoclonal bands).^8^ The most common CSF abnormality was the presence of CSF‐exclusive oligoclonal bands (*n* = 51 [of 80 tested, 64%]), followed by CSF pleocytosis ([>5/uL] *n* = 38 [of 88 tested, 43%], median value 13, range 6–392; lymphocytic predominant in 26 patients), elevated IgG index (*n* = 28 [of 74 tested, 38%]). A full list of CSF abnormalities is provided in Table 2.

### Oncologic associations

Fifty‐five patients (56%) had cancer. Neurologic symptoms preceded cancer detection in 38 of 55 patients with cancer (69%). The mean duration from symptom onset to cancer detection was 5 months (range, 0–90 months). The most frequent cancers were testicular germ cell tumor (*n* = 20; seminoma 19, nonseminomatous 1), followed by breast cancer (*n* = 9, ductal carcinoma 8) and small cell carcinoma (*n* = 6). A complete list of associated cancers is provided in Table 2. Three patients with KLHL‐11 autoimmunity who did not have detectable neoplasm had testicular microlithiasis Of the 55 patients with a cancer detected, 48 (87%) had cancers known to be associated with the neural antibody. In 7 patients, the tumor associations were atypical (Glycine‐R, 2 patients [chronic lymphocytic leukemia, testicular seminoma]; IgLON5, 1 patient [unclear histological origin]; KLHL, 1 patient [prostate adenocarcinoma]; AGNA1, 1 patient [lung adenocarcinoma]; leiomyosarcoma, 1 patient [ANNA‐1]; PCA‐1, 1 patient [Brenner tumor of ovary]).

### Antibody‐specific associations

Individual neural antibodies had neurologic phenotypic and oncologic associations. Compared with the remainder of the cohort, KLHL‐11‐IgG positivity associated with male sex (*p* ≤ 0.001), hearing loss (<0.001) and the presence of a testicular germ cell tumor (<0.001). The association between KLHL‐11‐IgG positivity and vestibulocochlear symptoms (tinnitus, vertigo) was also significant (*p* = 0.031). ANNA‐2‐IgG positivity was associated with jaw dystonia (*p* = 0.002) and laryngospasm (*p* = 0.033). Sleep disorders and chorea associated with IgLON‐5 autoimmunity (*p* < 0.001), whereas coexisting myelopathy associated with MOG‐IgG positivity (*p* = 0.049). A breakdown of antibody‐specific clinical associations is presented in Table S4.

### Treatments

All patients were treated with either immunotherapy or cancer treatment. The most frequent treatments administered included steroids (*n* = 89), IVIg (*n* = 61), plasma exchange (*n* = 48), and cyclophosphamide (*n* = 46). Forty‐six patients received cancer treatment (35 surgical tumor resection, 29 medical treatment). Following acute immunotherapy, 77 patients (79%) were prescribed maintenance immunotherapy (cyclophosphamide, 26; mycophenolate, 23; rituximab, 21; IVIg, 12; prolonged steroid taper, 7; intermittent plasmapheresis, 5; azathioprine, 5; ocrelizumab, 2; ibrutinib, 1).

### Treatment response

Treatment response was defined as subjective (when reported by the patient or recorded by provider) and objective (when there was an improvement in mRS score following treatment). Sixty‐two patients (63%) had a subjective treatment response. Twenty‐seven patients (28%) had an objective mRS change following treatment. Of 97 patients who received immunotherapy, 35 (36%) were completely immunotherapy refractory having neither an objective nor subjective response to treatment.

### Time to immunotherapy

Median time from neurologic symptom onset to immunotherapy initiation was 5 months (range, 0–138). Twenty‐one patients (22%) received immunotherapy within 1 month of symptom onset, and 42 (43%) within 3 months. Of patients who received immunotherapy within 1 month, 52% had an objective response to immunotherapy compared to 21% who received immunotherapy after 1 month (*p* = 0.011).

### Outcome

Outcome data (mRS at last follow‐up) were available for 97 of 98 patients (one patient was 17‐month‐old at last Mayo Clinic follow‐up and mRS was not applicable). The median duration of neurologic follow‐up for the cohort was 17 months (range, 0–213 months). At last follow‐up, 70 patients (71%) had mRS of >2 (mRS = 3, 30 patients; mRS = 4, 21 patients; mRS = 5, 11 patients; mRS = 6, 8 patients). The median mRS for the cohort at last follow‐up was 3. Eight patients died: 6 as a consequence of neurologic disorder; 2 had no data available. The median time from neurologic symptom onset to death was 20 months (range, 2–102 months).

Poor outcome was defined as mRS ≥4 at last Mayo Clinic follow‐up (*n* = 40 [41%]). Factors associated with poor outcome on univariate analysis are summarized in Table S5. On multivariate analysis, bulbar symptoms, abnormal MRI imaging, and elevated CSF IgG index were independently associated with poor outcome (Table 5).

### Cardiorespiratory compromise

Seven patients developed cardiac or respiratory compromise (cardiac arrest, 2; respiratory failure, 4; respiratory failure followed by cardiac arrest, 1). Dysphagia was present in all patients with cardiac or respiratory compromise (100% vs 58% of overall cohort, *p* = 0.041). Two of 5 patients with respiratory arrest had glycine receptor autoimmunity.

### Ambulatory outcomes

**Figure 3:**
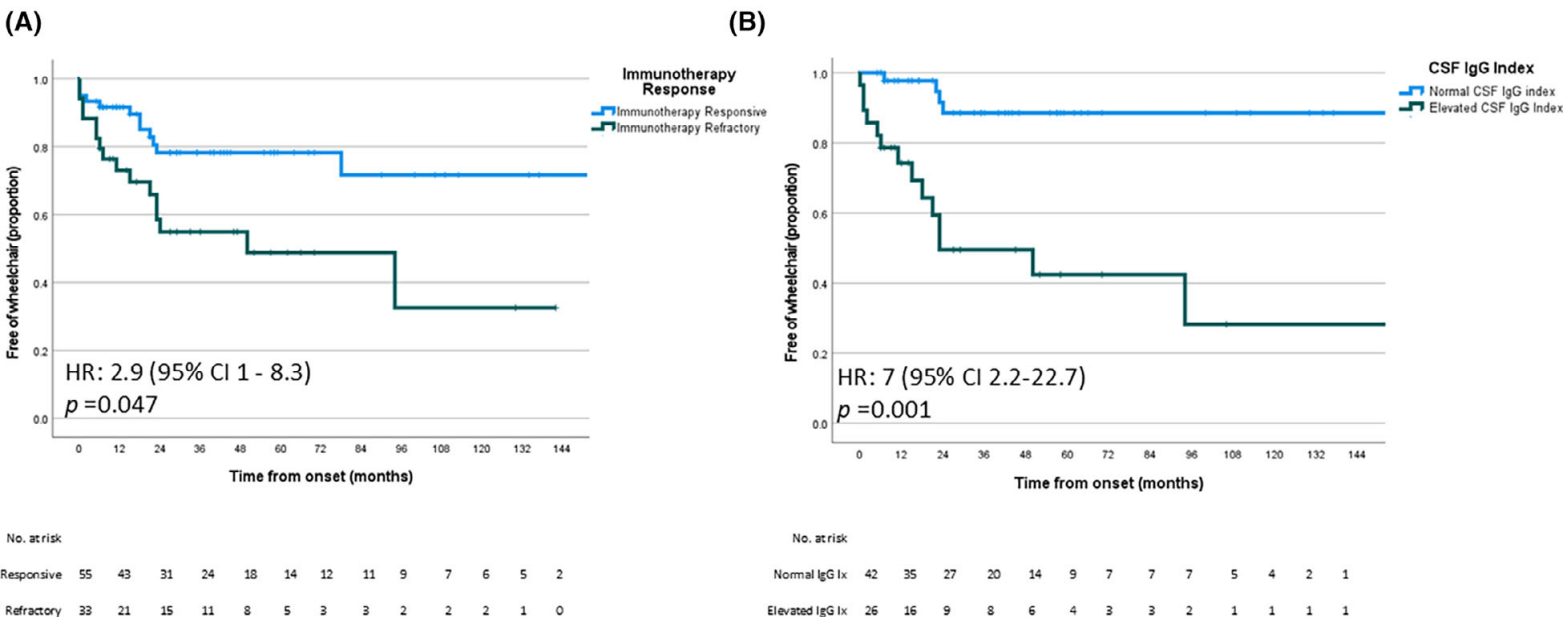
Kaplan–Meier curves estimating time to wheelchair dependence in patients with autoimmune brainstem encephalitis and subgroups. Progression to wheelchair dependence was faster among patients who were immunotherapy refractory (A), and patients with an elevated CSF IgG index (B).

Time from symptom onset to final ambulatory outcome was available for 97 patients. Final ambulatory outcomes were as follows: independently ambulatory (*n* = 28; 29%), ambulatory with gait aid (*n* = 38; 39% [cane *n* = 17, 45%; walker *n* = 21, 55%]), wheelchair dependent (*n* = 29; 30%), and bedbound (2; 2%). The time to wheelchair dependence (in 29 with data available) was estimated using Kaplan–Meier survival analysis. The median time to wheelchair dependence was 120 months. Patients who were immunotherapy refractory progressed to wheelchair dependence faster than those who responded to immunotherapy (*p* = 0.047). Among patients with an elevated CSF‐IgG index, 78% reached wheelchair dependence compared to only 22% with a normal CSF‐IgG index (*p* < 0.001). Patients with an elevated CSF‐IgG index progressed to wheelchair dependence faster than those with a normal CSF‐IgG index (*p* = 0.001). Kaplan–Meier curves estimating time to wheelchair dependence are shown in Figure 3.

### Diagnostic criteria

Nineteen patients (19%) met 2016 diagnostic criteria for autoimmune encephalitis.^2^ Fifteen of 19 patients met definite AE criteria on the basis of possible autoimmune encephalitis (presence of altered mental status, short‐term memory loss, or psychiatric symptoms in addition to brainstem symptomatology) and neural antibody positivity (GAD65, 5 patients [GAD65 and glycine receptor IgG 1 patient]; IgLON5, 4 patients; Ma2, 2 patients; KLHL‐11, 2 patients; ANNA1, DPPX, 1 patient each.); four patients fulfilled criteria for definite Bickerstaff's brainstem encephalitis (all were GQ1B antibody positive). The remaining 81% of the cohort did not have altered mental status, psychiatric symptoms, or cognitive deficits, and thus, existing AE criteria were not applicable. Proposed diagnostic criteria for definite and probable antibody‐negative autoimmune brainstem encephalitis are presented in Tables 3 and 4.

**Table 3:** Diagnostic criteria for definite autoimmune brainstem encephalitis.

Definite autoimmune brainstem encephalitis
Diagnosis can be made when all the following criteria are met: Onset^a^ of symptoms and signs localizing to the brainstem^b^ Presence of neural antibody^c^ associated with autoimmune brainstem encephalitis Reasonable exclusion of alternate causes^d^

^a^ Onset is subacute (<3 months to maximum deficit) in the majority of cases but indolent presentations may also occur.

^b^ Frequent localizing clinical features include diplopia (may also occur in isolated cerebellar disorder), ophthalmoplegia, bulbar dysarthria, dysphagia, vestibulocochlear symptoms (tinnitus, vertigo, hearing loss), nausea and vomiting, facial weakness, ptosis, and signs of trigeminal nerve dysfunction.

^c^ Amphiphysin; anti‐glial nuclear antibody (AGNA)‐1; ANNA‐1, ‐2 and ‐3; aquaporin‐4 (AQP4), collapsin‐response mediator protein (CRMP)‐5; contactin‐associated protein 2 [CASPR2]; dipeptidyl‐peptidase‐like protein (DPPX); glutamic acid decarboxylase 65 kDa isoform [GAD65] antibody [in the serum or any titer detected in the CSF]; glial fibrillary acidic protein (GFAP); glycine receptor; GTPase regulator associated with focal adhesion kinase (GRAF)‐1; GQ1B; IgLON‐5; inositol 1,4,5‐triphosphate receptor (ITPR)‐1; KLHL‐11; leucine‐rich glioma‐inactivated [LGI1] antibody; neurochondrin; neuronal intermediate filament (NIF), in CSF; N‐methyl‐D‐aspartate [NMDA] receptor [R] [in CSF]; Ma2; myelin oligodendrocyte glycoprotein (MOG); Purkinje cell cytoplasmic antibody (PCA)‐1 (anti‐Yo); PCA‐Tr; Phosphodiesterase 10A (PDE10A).

^d^ Alternate causes reasonable to exclude in the correct clinical context: infectious brainstem encephalitis, other inflammatory CNS disorders (Sarcoid, Behcet's, CLIPPERS, CNS vasculitis, multiple sclerosis); neoplastic disorders; drug toxicity; cerebrovascular disease; mitochondrial disorder, inborn errors of metabolism, nutritional deficiencies (e.g., Wernicke's encephalopathy).

**Table 4:** Diagnostic criteria for probable antibody‐negative autoimmune brainstem encephalitis.

Probable antibody‐negative autoimmune brainstem encephalitis
Diagnosis can be made when all the following criteria are met: Onset^a^ of symptoms and signs localizing to the brainstem^b^ Absence of characterized neural antibodies^c^ associated with brainstem encephalitis Both of the following: MRI findings^d^ suggestive of brainstem encephalitis Inflammatory CSF findings^e^ Reasonable exclusion of alternate causes^f^

^a^ Onset is subacute (<3 months to maximum deficit) in the majority of cases but indolent presentations may also occur.

^b^ Frequent localizing clinical features include diplopia (may also occur in cerebellar disorder), ophthalmoplegia, bulbar dysarthria, dysphagia, vestibulocochlear symptoms (tinnitus, vertigo, hearing loss), nausea and vomiting, facial weakness, ptosis, and signs of trigeminal nerve dysfunction.

^c^ Amphiphysin; anti‐glial nuclear antibody (AGNA)‐1; ANNA‐1, ‐2 and ‐3; aquaporin‐4 (AQP4), collapsin‐response mediator protein (CRMP)‐5; contactin‐associated protein 2 [CASPR2]; dipeptidyl‐peptidase‐like protein (DPPX); glutamic acid decarboxylase 65 kDa isoform [GAD65] antibody [in the serum or any titer detected in the CSF]; glial fibrillary acidic protein (GFAP); glycine receptor; GTPase regulator associated with focal adhesion kinase (GRAF)‐1; GQ1B; IgLON‐5; inositol 1,4,5‐triphosphate receptor (ITPR)‐1; KLHL‐11; leucine‐rich glioma‐inactivated [LGI1] antibody; neurochondrin; neuronal intermediate filament (NIF), in CSF; N‐methyl‐D‐aspartate [NMDA] receptor [R] [in CSF]; Ma2; myelin oligodendrocyte glycoprotein (MOG); Purkinje cell cytoplasmic antibody (PCA)‐1 (anti‐Yo); PCA‐Tr; Phosphodiesterase 10A (PDE10A).

^d^ MRI findings include T2 or FLAIR signal change in brainstem or cerebellum.

^e^ Inflammatory CSF is defined as: ≥1 CSF pleocytosis, CSF‐exclusive oligoclonal bands, elevated CSF IgG index, elevated CSF Kappa free light chains.

^f^ Alternate causes reasonable to exclude in the correct clinical context: infectious brainstem encephalitis, other inflammatory CNS disorders (Sarcoid, Behcet's, CLIPPERS, CNS vasculitis, multiple sclerosis); neoplastic disorders; drug toxicity; cerebrovascular disease; mitochondrial disorder, inborn errors of metabolism, nutritional deficiencies (e.g., Wernicke's encephalopathy).

**Table 5:** Factors associated with poor outcome following multivariate analysis.

Variable	*p* value	OR	95% CI lower	95% CI upper
Abnormal MRI brain	0.007*	9.46	1.85	48.32
Ataxia	0.57	1.73	0.26	11.52
Bulbar symptoms	0.005*	10.3	1.99	53.31
Diplopia	0.25	2.81	0.48	16.51
Elevated CSF IgG index	0.006*	8.02	1.82	35.4
Refractory to immunotherapy	0.056	0.24	0.05	1.03

CSF, cerebrospinal fluid; MRI, magnetic resonance imaging.

* *p* value <0.05.

### Validation of proposed criteria

All 98 included patients satisfied proposed diagnostic criteria for definite autoimmune brainstem encephalitis, which required the presence of a diagnostic neural antibody biomarker. A preliminary assessment of the sensitivity and specificity of probable antibody‐negative autoimmune brainstem encephalitis criteria was undertaken by reassessing the initial cohort of 633 patients who did not meet our inclusion criteria (Fig. S1). A clinical diagnosis of autoimmune brainstem encephalitis was used as gold standard. Of those 633 patients, 35 (6%) received a final clinical diagnosis of seronegative autoimmune brainstem encephalitis. Of those 35, 21 (60%) met criteria for probable antibody‐negative autoimmune brainstem encephalitis (sensitivity of 60%). Fourteen (40%) did not meet criteria due to ≥1 of: (i) lack of symptoms localizing to brainstem (*n* = 0), (ii) lack of MRI changes suggestive of autoimmune brainstem encephalitis (*n* = 8), (iii) lack of inflammatory CSF findings (*n* = 8), reasonable exclusion of alternate causes (*n* = 0).

Another 64 patients of the 633 (10%) were initially suspected to have seronegative autoimmune brainstem encephalitis, but had alternative final diagnoses (specificity of 100%). Forty‐eight (75%) did not meet criteria due ≥1 of: (i) lack of symptoms localizing to brainstem (*n* = 8), lack of MRI changes suggestive of autoimmune brainstem encephalitis (*n* = 24), and lack of inflammatory CSF findings (*n* = 39). Sixteen patients (25%) satisfied clinical, MRI and CSF requirements for probable autoimmune brainstem encephalitis but did not meet criteria due to the “reasonable exclusion of alternate causes” criterion. These alternate diagnoses were as follows: other inflammatory CNS disorders (*n* = 7 [multiple sclerosis, 2; ADEM, 1; Susac syndrome, 1; neurosarcoidosis, 1; acute flaccid myelitis, 1; Erdheim‐Chester disease, 1]) infectious brainstem encephalitis (*n* = 5 [West Nile Virus, 2; Streptococcus pneumoniae, 1; Scedosporium, 1; progressive multifocal leukoencephalopathy, 1]); neoplasm (*n* = 4 [lymphoma, 2; astrocytoma, 1; EBV lymphoproliferative disease, 1]).

## Discussion

We found autoimmune brainstem encephalitis to be more prevalent in men, in contrast to other autoimmune CNS disorders which have a female preponderance, likely influenced by KLHL‐11‐IgG as the leading serological finding.^9^, ^10^ Symptom onset was preceded by a low‐grade fever or flu‐like prodrome in 21% of cases and thus is not helpful in distinguishing autoimmune from infectious etiologies.^11^ No patients had a history of immune checkpoint inhibitor use, but such cases are commonly seronegative.^12^ Symptom onset was typically subacute (<3 months until maximal deficit); however, 18% of cases were indolent. Specifically, a chronic disease course occurred in all cases of anti‐IgLON‐5 disease and in one third of KLHL‐11 cases.

Higher cortical signs (altered mental status, seizures, psychiatric) were infrequent. Rather, the most frequent clinical presentations included: diplopia, vertical nystagmus, facial weakness, vestibulocochlear dysfunction (vertigo, tinnitus or hearing loss), or bulbar symptoms. Nausea and vomiting were common overall and occurred as the presenting symptom in 7%. Cerebellar ataxia co‐existed in the majority, which is not surprising given the anatomic juxtaposition and functional integration between brainstem and cerebellum. In the absence of brainstem dysfunction, autoimmune cerebellar ataxia is a separate disorder, the phenotype of which is well characterized.^13^, ^14^ Differentiating autoimmune brainstem encephalitis from autoimmune cerebellar ataxia can be challenging when diplopia is the only symptom referrable to the brainstem. Diplopia alone, without evidence of ophthalmoparesis or skew deviation, seems insufficient for a diagnosis of autoimmune brainstem encephalitis.^15^ Similarly, other isolated clinical presentations (e.g., isolated nystagmus or vestibulocochlear dysfunction) in the absence of other symptoms or signs localizing to the brainstem should be interpreted with caution.

Consistent with the critical role of the brainstem in sleep regulation, a wide spectrum of sleep disorders occurred in patients with autoimmune brainstem encephalitis, most frequently sleep apnea and insomnia. It is notable that following ataxia, the most frequent movement disorder among patients with autoimmune brainstem encephalitis was dystonia. Interaction between the basal ganglia and the brainstem, in particular the midbrain and superior colliculus, has been implicated in the pathogenesis of primary dystonias.^16^, ^17^ Less common clinical manifestations of autoimmune brainstem encephalitis – but noteworthy due to their localizing potential – included laryngospasm, palatal myoclonus, and hiccups. Some clinical features may serve as bedside clues to the underlying neural antibody specificity in the correct clinical context (e.g., hearing loss and KLHL‐11 autoimmunity).

The diagnostic work‐up of patients with autoimmune encephalitis should include MRI imaging, CSF studies, neural antibody screening and – in the presence of high‐ or intermediate‐risk paraneoplastic antibodies – a diligent search for occult cancer.^18^ It is notable that the most frequent CSF abnormality identified was the presence of CSF‐exclusive oligoclonal bands and this test should therefore be requested in suspected cases. However, the absence of inflammatory CSF or MRI abnormalities does not exclude a definite diagnosis of autoimmune brainstem encephalitis. For example, only one third of patients had signal change within the brainstem. Other imaging abnormalities such as brainstem/cerebellar atrophy or hypertrophic olivary degeneration are late findings which are not specific for an inflammatory etiology. In the correct clinical context, the presence of validated disease‐specific neural antibody biomarkers associated with autoimmune brainstem encephalitis is the key diagnostic test in arriving at a definite diagnosis.

We found diverse disease‐specific neural antibodies (21 IgGs in total) associated with autoimmune brainstem encephalitis, optimally detected when serum and CSF tests were combined. These neural antibodies should inform the profile of requested analytes where there is clinical suspicion for an immune‐mediated brainstem disorder. A notable absence among our cohort is GFAP‐IgG which has been recently reported to present with features of rhombencephalitis and should therefore also be considered in the evaluation of autoimmune brainstem disorders.^19^ In patients with >1 neural antibody, features associated with each antibody specificity often occurred (e.g., a male patient with ANNA‐2 and KLHL‐11‐IgGs presented with jaw dystonia [most commonly ANNA‐2 associated] and an underlying testicular germ cell tumor [KLHL‐11 associated]), emphasizing the clinical utility of comprehensive neural antibody evaluations in patient management.

Over half of patients within our cohort had a paraneoplastic autoimmune brainstem encephalitis. Testicular cancer was the most frequent neoplasm emphasizing the need for testicular imaging in the cancer screening of patients with suspected immune‐mediated brainstem disorders. In autoimmune paraneoplastic neurologic disorders, neural antibodies inform the risk of malignancy and the likely histological subtype.^20^ For example, of the 21 patients with testicular malignancy, only 2 neural antibodies accounted for the vast majority of cases (KLHL‐11‐IgG: 15 cases; Ma2‐IgG: 4 cases). Neurologic symptoms preceded cancer identification in most patients, highlighting that comprehensive neural antibody evaluations may facilitate early cancer detection.

Syndrome‐based diagnostic criteria proposed in 2016 have been helpful for assessment of patients with suspected AE where altered mental status is a cardinal finding, including a minority of patients with brainstem encephalitis (namely Bickerstaff encephalitis), but otherwise are not designed to assess patients with suspected autoimmune brainstem encephalitis.^1^, ^2^ We found similar results in our cohort with only 14 patients (14%) meeting clinical criteria for possible autoimmune encephalitis, most of whom had multifocal neurologic disorders with additional supratentorial signs.

Updated diagnostic criteria for paraneoplastic neurologic disorders have incorporated the presence of neural antibody biomarkers to arrive at a definite diagnosis.^18^ Similarly, attempts have been made to incorporate neural antibodies into existing autoimmune encephalitis criteria, especially for patients who do not meet the clinical requirements for diagnosis (either due to indolent disease onset or paucisymptomatic presentations).^21^ We have adopted a similar approach here, proposing brainstem encephalitis criteria, emphasizing clinical and standard neurologic test results in the first instance, and disease‐specific neural antibodies to assist in reaching a definite diagnosis. Additionally, our criteria allow for a more standardized nomenclature when categorizing neural antibody‐associated autoimmune brainstem disorders (as opposed to, for example, eponymous nomenclature). Clinical localization to the brainstem, in addition to inflammatory‐appearing MRI and CSF are required to reach a diagnosis of “probable autoimmune brainstem encephalitis,” but it is critically important to ensure exclusion of other previously characterized inflammatory disorders, infectious or neoplastic etiologies. Our initial assessment indicates these criteria could be applied in clinical practice, but require further validation in an independent cohort.

Patient outcomes overall were poor. Elevated CSF‐IgG index was independently associated with both poorer outcomes and faster progression to wheelchair dependence. Interestingly, elevated CSF‐IgG index has been previously reported to predict worse neurologic outcomes in infectious CNS disease, but this is the first instance we are aware of elevated CSF‐IgG index portending poor prognosis in autoimmune neurologic disorders.^22^ No neural antibody specificity reached statistical significance in associating with poor outcome; however, it is noteworthy that all four patients with PCA‐1‐IgG had poor outcome at last follow‐up (two died, and two required constant nursing care [mRS = 5]).

Bulbar symptoms were the only independent clinical symptom associating with poor outcome; it is notable that dysphagia (implicating medullary involvement) was present in all seven patients who suffered cardiac or respiratory compromise. Of four patients with glycine receptor‐IgG‐associated brainstem encephalitis, two patients developed respiratory failure (one of whom died). A third patient with glycine receptor‐IgG‐associated brainstem encephalitis in our cohort had laryngospasm and shortness of breath. In a prior series of patients with glycine receptor autoimmunity, brainstem involvement was a prominent feature and, although most patients were immunotherapy responsive, there was a 9% mortality rate.^23^

Short time to immunotherapy is associated with improved outcomes in autoimmune neurologic disorders.^24^, ^25^ We found a significant difference in the number of patients who objectively improved (decrease in mRS score) following administration of immunotherapy <1 month after symptom onset compared to those who received immunotherapy >1 month following symptom onset. These findings emphasize that early initiation of immune treatment offers the highest likelihood for objective neurologic improvement.

Limitations of this study include retrospective study design for assessment of neurologic outcome. Inclusion of patients from a tertiary referral center may lead to an overrepresentation of severe or relapsing cases, or patients who are late in disease course, thus limiting the generalizability of our findings. Similarly, the discovery of KLHL‐11‐IgG at our institution may have contributed to its high representation in our cohort. Use of mRS may have been insensitive to nonmotor disability. It is possible that some patients were overlooked in our search because the inclusion diagnoses were restricted to brainstem encephalitis and rhombencephalitis.

Overall, this study defines the neurologic phenotypic spectrum of autoimmune brainstem encephalitis, its neural antibody and oncologic associations, in addition to factors associated with poor outcome. Proposed diagnostic criteria will aid in diagnosis of this subphenotype of autoimmune encephalitis. Future studies should validate factors associated with poor outcome, the range of neural antibodies associated with brainstem encephalitis, and our proposed diagnostic criteria.

## Author Contributions

Conception and design of the study: AM. Acquisition and analysis of data: all authors. Drafting a significant portion of the manuscript or figures: MG and AM.

## Conflict of Interest

MG has a patent pending for CAMKV‐IgG. ST reports no disclosures relevant to the manuscript. EO reports no disclosures relevant to the manuscript. SB reports no disclosures relevant to the manuscript. EPF: Dr Flanagan has served on advisory boards for Alexion, Genentech, Horizon Therapeutics, and UCB. He has received research support from UCB. He received royalties from UpToDate. Dr Flanagan is a site principal investigator in a randomized clinical trial of Rozanolixizumab for relapsing myelin oligodendrocyte glycoprotein antibody‐associated disease run by UCB. Dr Flanagan is a site principal investigator and a member of the steering committee for a clinical trial of satralizumab for relapsing myelin oligodendrocyte glycoprotein antibody‐associated disease run by Roche/Genentech. Dr Flanagan has received funding from the NIH (R01NS113828). Dr Flanagan is a member of the medical advisory board of the MOG project. Dr Flanagan is an editorial board member of Neurology, Neuroimmunology and Neuroinflammation, The Journal of the Neurological Sciences and Neuroimmunology Reports. A patent has been submitted on DACH1‐IgG as a biomarker of paraneoplastic autoimmunity. SLC reports no disclosures relevant to the manuscript. JMT reports no disclosures relevant to the manuscript. JRM reports no disclosures relevant to the manuscript. SJP has received personal compensation for serving as a consultant for Roche/Genentech, Sage Therapeutics, Arialys, and Astellas. He's received personal compensation for serving on scientific advisory boards or data safety monitoring boards for F. Hoffman‐LaRoche AG, Genentech, Arialys, and UCB. His institution has received compensation for serving as a consultant for Astellas, Alexion, and Viela Bio/MedImmune. All compensation is paid to Mayo Clinic. He has received research support from Alexion, Viela Bio/MedImmune, Roche/Genentech, and Adimmune. He has a patent, Patent #8,889,102 (Application #12‐678350, Neuromyelitis Optica Autoantibodies as a Marker for Neoplasia) – issued; a patent, Patent #9,891,219B2 (Application #12‐573942, Methods for Treating Neuromyelitis Optica (NMO) by Administration of Eculizumab to an individual that is Aquaporin‐4 (AQP4)‐IgG Autoantibody positive)‐issued and from which he has received royalties and a patent for GFAP‐IgG; Septin‐5‐IgG; MAP1B‐IgG; Kelch‐like protein 11; PDE10A pending. He is working as a consultant in the Mayo Clinic Neuroimmunology laboratory clinical service. The Mayo Clinic Neuroimmunology Laboratory commercially offers testing, but revenue accrued does not contribute to salary, research support, or personal income. CVS reports no disclosures relevant to this manuscript. AZ has patent applications pending on CAMKV‐IgG, PDE10A‐IgG, and DACH1‐IgG as biomarkers of paraneoplastic neurological autoimmunity and has received research funding from Genentech. DD has research support from Department of Defence (CA210208), Centers of Multiple Sclerosis and Autoimmune Neurology, and Clinical and Translational Science, Mayo Clinic, and Grifols pharmaceuticals, has consulted for UCB, Immunovant, Argenx, and Astellas pharmaceuticals (compensation for consulting activities paid directly to Mayo Clinic), and has patents pending for KLHL11‐IgG, LUZP4‐IgG and cavin‐4‐IgG as markers of neurological autoimmunity. AM reports research funding from National Institutes of Health (NIH: RO1NS126227, U01NS120901), patents issued for GFAP and MAP1B‐IgGs and patents pending for CAMKV, PDE10A, Septins‐5 and ‐7, and KLHL11‐IgGs, and has consulted for Janssen and Roche pharmaceuticals, without personal compensation.

## Supporting information


Figure S1.

Table S1.

Caption S1.

## Data Availability

Anonymized data used for this study are available on request.
