# Supplementary figures and images for: Autoimmune brainstem encephalitis: Clinical associations, outcomes, and proposed diagnostic criteria
Source: Ann Clin Transl Neurol. 2024 Dec 21;12(1):213–25. doi: 10.1002/acn3.52273 (PMC11752097; doi:10.1002/acn3.52273)

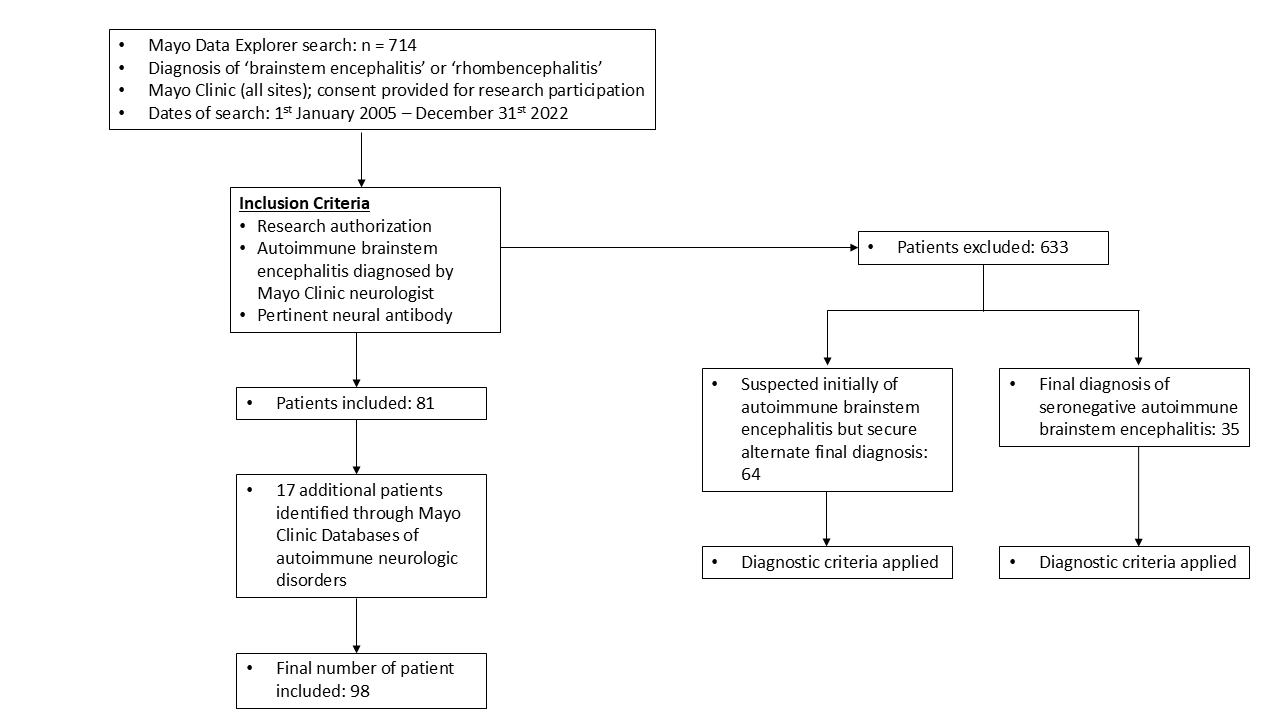

Supplement: Supplementary file 1 — Figure S1. [file ACN3-12-213-s003.tif]
