# Supplementary material for: Autoimmune brainstem encephalitis: Clinical associations, outcomes, and proposed diagnostic criteria
Source: Ann Clin Transl Neurol. 2024 Dec 21;12(1):213–25. doi: 10.1002/acn3.52273 (PMC11752097; doi:10.1002/acn3.52273)
Supplement: Supplementary file 2 — Table S1. [file ACN3-12-213-s001.docx]

**Supplementary Material for Review and Publication


Supplementary Table 1. Movement and sleep disorder frequency in autoimmune brainstem encephalitis**

| **Movement disorders** | **No. (%)** |
| --- | --- |
| Any movement disorder | 86 (88) |
| Ataxia | 76 (78) |
| Any dystonia  Jaw dystonia | 17 (17)  9 (9) |
| Postural instability | 16 (16) |
| Parkinsonism | 8 (8) |
| Opsoclonus Myoclonus | 6 (6) |
| Stiff person spectrum disorder | 5 (5) |
| Laryngospasm | 4 (4) |
| Palatal myoclonus | 4 (4) |
| Chorea | 3 (3) |
| **Sleep disorders** | **No. (%)** |
| Any sleep disorder | 28 (29) |
| Sleep apnea (central) | 12 (12) |
| Insomnia | 10 (10) |
| REM sleep behavior disorder | 10 (10) |
| Hypersomnia | 6 (6) |
| Cataplexy | 3 (3) |
| Narcolepsy | 2 (2) |

*REM: rapid eye movement* **Supplementary Table 2. Frequency of neural antibodies**

| **Neural Antibody** | **No. (%)** |
| --- | --- |
| KLHL-11* | 26 (27) |
| GAD-65** | 12 (12) |
| ANNA-1 | 8 (8) |
| ANNA-2* | 8 (8) |
| Ma2 | 7 (7) |
| IgLON-5 | 6 (6) |
| AQP4* | 6 (6) |
| MOG | 4 (4) |
| Glycine-R* | 4 (4) |
| GQ1B* | 4 (4) |
| PCA-1 | 4 (4) |
| NIF* | 2 (2) |
| NMDA-R** | 2 (2) |
| DPPX | 2 (2) |
| Neurochondrin | 2 (2) |
| AGNA-1 | 1 (1) |
| Amphiphysin | 1 (1) |
| ANNA-3* | 1 (1) |
| CRMP-5 | 1 (1) |
| ITPR-1 | 1 (1) |
| PCA-2 | 1 (1) |
| PDE10A | 1 (1) |

*Each asterisk represents a single patient with multiple antibodies
*KLHL-11: Kelch-like-protein 11; GAD: glutamic acid decarboxylase; ANNA: antineuronal nuclear antibody; AQP4: aquaporin 4; MOG: myelin oligodendrocyte glycoprotein; PCA: Purkinje cell cytoplasmic antibody; NIF: neuronal intermediate filament; NMDA-R: N-methyl-D-aspartate; DPPX: dipeptidyl-peptidase-like protein; AGNA: anti-glial nuclear antibody; CRMP: collapsin-response mediator protein; ITPR-1: inositol 1, 4, 5-triphosphate receptor 1; PDE: phosphodiesterase inhibitor.*

**Supplementary Table 3. Clinical features of patients with multiple neural antibodies**

| Presence of multiple IgGs | Neurologic phenotype | Cancer |
| --- | --- | --- |
| ANNA-2 + KLHL-11 | Vertigo, ataxia, jaw dystonia, sleep disorder and respiratory failure requiring mechanical ventilation | Testicular GCT |
| AQP4 + NMDA-R | Atypical demyelinating brainstem lesions, double vision, and encephalopathy | N/A |
| GAD65 + GQ1B + NMDA-R | GI prodrome, nausea and vomiting, ataxia, diplopia, dysphagia, with encephalopathy and short-term memory loss | N/A |
| ANNA-3 + NIF | Vertigo, vertical nystagmus, diplopia, and cerebellar ataxia | SCLC |
| GAD65 + Glycine-R | Multiple cranial neuropathies with encephalopathy, choking spells, myoclonus, hyperekplexia and respiratory arrest | N/A |

*ANNA: antineuronal nuclear antibody; KLHL-11: kelch-like protein 11; AQP4: aquaporin 4; NMDA-R: n-methyl-D-aspartate receptor; GAD: glutamic acid decarboxylase; NIF: neuronal intermediate filament.* **Supplementary Table 4
Neural antibody clinical associations**

| **Clinical feature** | **Antibody association** | **Antibody-positive group with clinical feature (%)** | **Remainder of cohort with clinical feature (%)** | **p-value** |
| --- | --- | --- | --- | --- |
| Male | KLHL-11 Ma2 | 26 (100) 7 (100) | 30 (42) 49 (54) | <0.001 0.019 |
| Female | AQP4 PCA-1 | 6 (100) 4 (100) | 36 (39) 38 (40) | 0.013 0.031 |
| Hearing loss | KLHL-11 | 15 (58) | 8 (11) | <0.001 |
| Vestibulocochlear symptoms | KLHL-11 | 22 (85) | 44 (61) | 0.031 |
| Co-existing myelopathy | MOG | 3 (75) | 17 (18.1) | 0.026 |
| Brainstem signal change | MOG AQP4 | 4 (100) 6 (100) | 26 (28) 24 (26) | 0.008 <0.001 |
| Opsoclonus myoclonus syndrome | ANNA-2 | 4 (50) | 2 (2) | <0.001 |
| Jaw dystonia | ANNA-2 | 4 (50) | 5 (6) | 0.002 |
| Laryngospasm | ANNA-2 | 2 (25) | 2 (2) | 0.033 |
| Respiratory arrest | Glycine-R | 2 (50) | 3 (3) | 0.012 |
| Sleep disorder | IgLON5 | 6 (100) | 22 (24) | <0.001 |
| Chorea | IgLON5 | 3 (50) | 0 (0) | <0.001 |
| Cataplexy/Narcolepsy | Ma2 | 2 (29) | 1 (1) | 0.013 |
| Testicular germ cell tumor | KLHL-11 Ma2 | 15 (58) 4 (57) | 5 (7) 16 (18) | <0.001 0.031 |

*KLHL-11: Kelch like protein 11; AQP4: aquaporin 4; PCA: Purkinje cell cytoplasmic antibody; MOG: myelin oligodendrocyte glycoprotein; ANNA: antineuronal nuclear antibody.*

**Supplementary Table 5.
Variables associated with poor outcome (mRS ≥4) on univariate analysis.**

| **Variable*** | **Other outcome** | | **Poor outcome** | | **p-value** | **OR** | **95% CI lower** | **95% CI upper** |
| --- | --- | --- | --- | --- | --- | --- | --- | --- |
|  | No. | Percent | No. | Percent |  |  |  |  |
| Abnormal brain MRI | 31 | 54.4% | 31 | 77.5% | 0.02 | 2.889 | 1.166 | 7.155 |
| Ataxia | 40 | 70.2% | 36 | 90% | 0.02 | 3.825 | 1.177 | 12.430 |
| Bulbar symptoms | 29 | 50.9% | 35 | 87.5% | <0.001 | 6.759 | 2.315 | 19.730 |
| Cerebellar atrophy | 9 | 15.8% | 18 | 45% | 0.003 | 4.364 | 1.694 | 11.238 |
| CN XII palsy | 3 | 5.3% | 8 | 20% | 0.47 | 4.5 | 1.113 | 18.195 |
| CSF OCBs | 25 | 51% | 25 | 83% | 0.004 | 4.8 | 1.579 | 14.588 |
| Diplopia | 41 | 71.9% | 37 | 92.5% | 0.012 | 4.813 | 1.298 | 17.851 |
| Dysarthria (cerebellar or bulbar) | 30 | 52.6% | 37 | 92.5% | <.001 | 11.1 | 3.067 | 40.176 |
| Dysphagia | 29 | 50.9% | 30 | 75% | 0.017 | 2.897 | 1.196 | 7.013 |
| Elevated CSF IgG index | 11 | 22.9% | 16 | 64% | <.001 | 5.980 | 2.075 | 17.230 |
| Gaze palsy | 20 | 35.1% | 21 | 52.5% | 0.99 | 2.045 | 0.869 | 4.667 |
| Immunotherapy-refractory | 13 | 23.2% | 21 | 53.8% | 0.002 | 3.859 | 1.595 | 9.339 |

*CN: cranial nerve; CSF: cerebrospinal fluid; OCB: oligoclonal band;*
*Variables tested which were not significant on univariate analysis included (p > 0.1): sex, age, ptosis, symptoms of trigeminal nerve dysfunction, hearing loss, movement disorder, sleep disorder, presence of cancer, inflammatory CSF, CSF pleocytosis, presence of multiple antibodies, presence of neural antibody specificity (ANNA-1, ANNA-2, AQP4, GAD-65, Glycine-R, GQ1B, IgLON5, KLHL-11, Ma2, MOG)

**Supplementary Table 6. Pre-existing personal history of autoimmunity**

| **Autoimmune condition** | **No.(% of entire cohort)** |
| --- | --- |
| All autoimmune conditions | 24 (25%) |
| **Non-neurologic autoimmunity** |  |
| Autoimmune Thyroid Disease | 8* |
| Inflammatory Bowel Disease | 3* |
| Type 1 Diabetes Mellitus | 3* |
| Psoriasis | 2 |
| Sjrogen’s syndrome | 2* |
| Autoimmune corneitis | 1 |
| Autoimmune Polyendocrine Syndrome | 1 |
| Primary Biliary Cirrhosis | 1* |
| Pyoderma Gangrenosum | 1* |
| Raynaud’s | 1 |
| Systemic Lupus Erythematosus | 1 |
| Vitiligo | 1 |
| **Neurologic Autoimmunity** |  |
| Chronic Inflammatory Demyelinating Neuropathy | 1 |
| Myasthenia Gravis | 1 |

*Each asterisk represents a patient with multiple autoimmune conditions
