# Supplementary material for: Autoimmune brainstem encephalitis: Clinical associations, outcomes, and proposed diagnostic criteria
Source: Ann Clin Transl Neurol. 2024 Dec 21;12(1):213–25. doi: 10.1002/acn3.52273 (PMC11752097; doi:10.1002/acn3.52273)
Supplement: Supplementary file 3 — Caption S1. [file ACN3-12-213-s002.docx]

**Caption for Supplementary Figure 1

Supplementary Figure 1:** Study inclusion algorithm
